# Finite Element Simulation Study of Ultrasonic Vibration-Assisted Tensile High-Volume Fraction SiCp/Al Composite

**DOI:** 10.3390/ma12233841

**Published:** 2019-11-21

**Authors:** Zhi-meng Zhang, Dao-hui Xiang, Bang-fu Wu, Hao-ren Feng, Zhan-li Shi, Yong-wei Hu, Bo Zhao

**Affiliations:** School of Mechanical and Power Engineering, Henan Polytechnic University, Jiaozuo 454000, Henan, China

**Keywords:** ultrasonic vibration, tensile, SiCp/Al composite, Abaqus finite element simulation

## Abstract

Silicon carbide particle-reinforced aluminum matrix composite (SiCp/Al) has been widely used in the military and aerospace industry due to its special performance; however, there remain many problems in the processing. The present paper introduces an ultrasonic vibration tensile apparatus and a composite tensile specimen and performs Abaqus finite element simulation on high-volume SiCp/Al. The results show that the stress-strain curve increases linearly during conventional tensile strength; the intermittent vibration tensile strength is similar to the full course vibration tensile strength: The magnitude of the stress reduction increases as the amplitude of the ultrasound increases and the vibration frequency increases. The tensile rate is inversely proportional to the magnitude of the stress reduction, and in the ultrasonic parameters, the amplitude has the greatest influence on the magnitude of the stress reduction, followed by the tensile rate; additionally, the frequency has the least influence on the magnitude of the stress reduction. The experimental results show that the simulation results are consistent with the experimental results.

## 1. Introduction

Metal matrix composites (MMCs) have generated considerable recent research interest [1,2,3,4]. SiCp/Al composite is a special material prepared using aluminum alloy as a metal matrix followed by the addition of a quantitative volume fraction or different sizes of silicon carbide particles as reinforcement. SiCp/Al composite has been widely used in military, aerospace, transportation, and optical instruments due to its high specific strength and stiffness, as well as its wear resistance, low thermal expansion coefficient, and other advantages [5,6,7]. For SiCp/Al composites, many scholars have explored processing methods. Xu et al. [8] studied the friction and wear properties of CVD diamond-coated tools in the processing of SiCp/Al composites. It was found that diamond-coated tools can be used to process SiCp/Al composites under specific processing conditions, but the economic benefits are relatively poor. Sachin et al. [9] tested the SiCp/Al composite by EDM. The results show that EDM has a long time and low efficiency. With the increase of the SiC particle volume fraction, the material removal rate will be further reduced, and the surface roughness will increase. Ge et al. [10] studied the chip formation mechanism of SiCp/Al (15%) composites by precise turning tests. The research shows that the enhanced particle body ratio, cutting speed, feed rate and tool edge radius are the main factors affecting the chip formation. 

However, there are different mechanical properties between the matrix and the reinforcing material in the SiCp/Al composite, which makes the SiCp/Al composite still have many problems in conventional processing. Therefore, compound processing technology, especially ultrasonic vibration machining technology, has been proposed to solve these problems. Zha et al. [11] compared the removal mechanism of SiCp/Al composites under conventional and ultrasonic vibration conditions via a scratch test. It was found that the ultrasonic vibration reduced the friction coefficient between the material and the cutter, and the adhesion between the matrix and the SiCp was weakened, helping the removal of SiC particles; Wei et al. [12] proposed ultrasonic-assisted turning (UAT) to process SiCp/Al composites. Compared to conventional turning (CT) with ultrasonic assistance, the cutting force is significantly reduced, and the cutting temperature is slightly increased. But whether one uses UAT or CT, the tool results in abrasive wear and adhesive wear; Zhou et al. [13] used rotary ultrasonic grinding to process SiCp/Al materials and found that ultrasonic vibration can effectively reduce the grinding force, improving the material removal efficiency and surface quality; additionally, they did not observe the grinding wheel blockage and grinding burn phenomenon; in Wei et al. [14], the machinability and tool wear of machining a SiCp/Al metal matrix-composite was compared with dry UAT and CT with the use of a cemented carbide (WC) and a polycrystalline diamond (PCD) tool; they found that the cutting force was significantly reduced during UAT. The machined surface obtained in UAT with a WC tool was comparable and sometimes even better than that achieved with the PCD tool.

According to the existing research, the main obstacle to the wide application of MMCs is the maintenance of mechanical properties and material properties of processed materials [15,16,17]. Therefore, improving the processing quality of MMCs and maintaining the mechanical properties and material properties after processing have become a popular research area [18]. Although the technology of ultrasonic vibration composite machining has improved significantly in terms of tool wear, machining efficiency, and surface quality, there are still many inherent mechanisms that remain unclear. Therefore, studying the mechanical properties of high-strength SiCp/Al composites under ultrasonic vibration excitation has become an urgent problem to be solved.

In the present article, we designed an ultrasonic vibration tensile device and composite samples. The finite element simulation of the ultrasonic vibration tensile strength for high-volume fraction SiCp/Al composites was carried out, and the results of the simulation and experiment were consistent. The influence of the ultrasonic amplitude and frequency on the magnitude of the stress reduction was analyzed. It was found that there is a positive correlation between the frequency and amplitude, and the magnitude of the stress reduction; the tensile rate is inversely proportional to the magnitude of the stress reduction. The influence of the ultrasonic parameters on the magnitude of the stress reduction via the finite element simulation was that the amplitude had the greatest influence on the stress reduction amplitude, followed by the tensile rate; additionally, the frequency variation had the least influence on the stress reduction amplitude.

## 2. Ultrasonic Vibration Tensile Horn Design 

**Figure 1:**
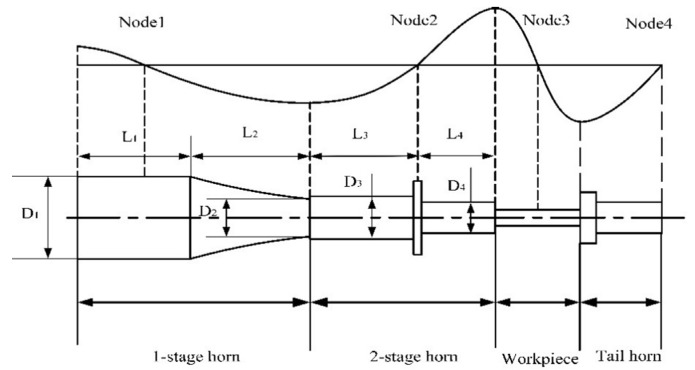
Schematic diagram of the horn.

The horn design is shown in Figure 1. The first-stage horn is a composite catenary horn with a wide-terminated cylinder, while the second-stage horn is a step-shaped horn, and the tail horn is a 1/2 wavelength co-frequency horn. The experimental design frequency was 20 kHz and 28 kHz, respectively. These materials were 45 steel. The related parameters are shown in Table 1. 

**Figure 2:**
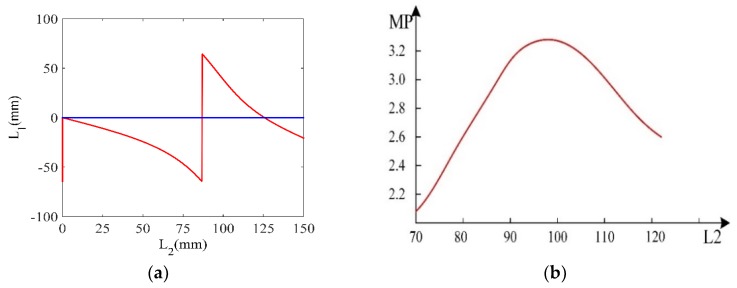
(**a**) Relationship between $L_{1}$ and $L_{2}$; (**b**) Relationship between $M_{P}$ and $L_{2}$.

When the frequency was 20 kHZ, the diameter of the first-stage horn was: $D_{1}=52\;\mathrm{mm},D_{2}=24\;\mathrm{mm}$. The resonant frequency and amplification factor equations based on the composite catenary horn [19] are:

(1) $$\tan\left(kl_{1}\right)=-\frac{\sqrt{k^{2}-\gamma^{2}}}{k}\tan\left(l_{2}\sqrt{k^{2}-\gamma^{2}}\right)-\frac{\gamma }{k}\tanh\left(\gamma l_{2}\right),$$

(2) $$M_{p}=\left|N\frac{\cos\left(kl_{1}\right)}{\cos\left(\sqrt{{\left(kl_{2}\right)}^{2}-{\left(\cosh^{-1}N\right)}^{2}}\right)}\right|,$$

where $\gamma =\cosh^{-1}\left(N/l_{2}\right)$ and $N=D_{1}/D_{2}$. Bring the relevant parameters into the equation to get the relationship of $L_{1}$, $L_{2}$, $M_{P}$, as shown in Figure 2a,b. It is necessary to ensure that $L_{1},\;L_{2}$ are positive and $M_{P}$ is the largest. The relevant parameters of the first-order horn when the frequency is 20 kHZ are shown in Table 2. Similarly, according to $D_{3}=38\;\mathrm{mm},D_{4}=24\;\mathrm{mm}$, the relevant parameters of the first-order horn can be obtained at 28 kHZ, as shown in Table 2.

Similarly, the relevant parameters of the 2nd horn in 20 kHZ and 28 kHZ are shown in Table 3.

According to the gravitational wave calculation method, a composite dumbbell-shaped tensile specimen satisfying the resonance condition is designed [20,21,22]. The small end of the sample is a high-part SiCp/Al composite material, and the large end is 45 steel to reduce the processing difficulty. The resonance equation for a composite dumbbell-shaped tensile specimen composed of different materials is as follows:

(3) $$\tan\left(k_{1}x_{1}\right)\tan\left(k_{2}x_{2}\right)=\frac{\rho_{1}c_{1}r_{1}^{2}}{\phi \rho_{2}c_{2}r_{2}^{2}}.$$

The loss factor ϕ = 0.8 [23] is introduced due to the difference of material at the large and small ends and the connection of the threads. Where $\rho_{1}=3040\;\mathrm{kg}\cdot m^{3}$, $\rho_{2}=7850\;\mathrm{kg}\cdot m^{3}$, $c_{1}=9212m/s$, $c_{2}=5170m/s$. k=ω/c, ω=2πf. The size of the model to be substituted into the relevant parameters is shown in Table 4.

**Figure 3:**
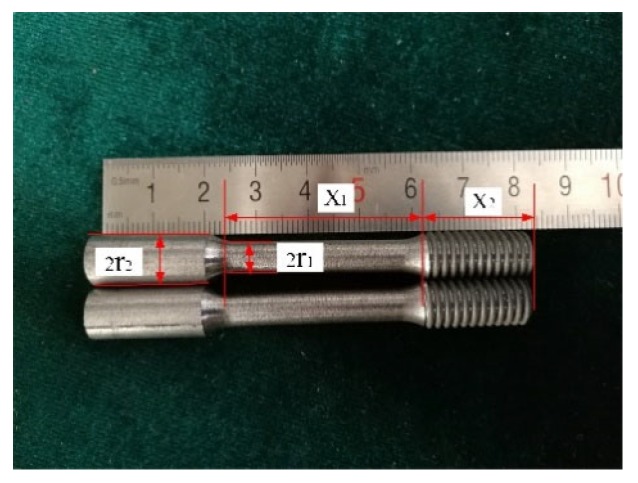
Tensile specimen of the SiCp/Al composite.

**Figure 4:**
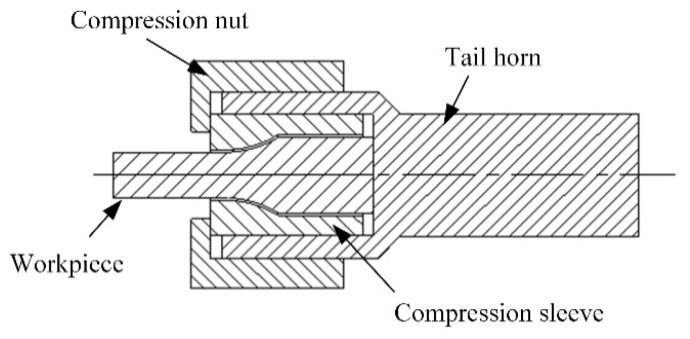
Connecting structure diagram of the sample and tail horn.

The sample has a minimum diameter of 6 mm and a gauge length of 30 mm, as shown in Figure 3. The tail horn connection is shown in Figure 4: the compression sleeve was cut from the middle of the axis to enclose the arcuate transition of the workpiece. The inner wall of the outer wall of the compression sleeve and the rear end horn were clearance-fitted and fixed by a compression nut. To make the resonant frequency of the system closer to the theoretical design frequency, the sample and the end horn were assembled for a modal analysis according to the size of the sample. The frequency is close to 20 kHZ and 28 kHZ to obtain the tail horn size.

**Figure 5:**
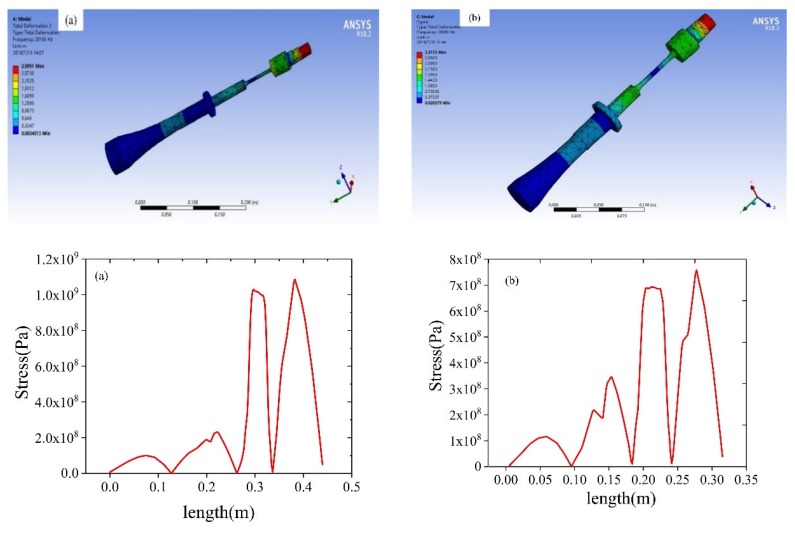
Simulation analysis of the ultrasonic vibration tensile resonance system (**a**) 20 KHz; (**b**) 28 KHz.

The ANSYS modal analysis of the ultrasonic vibration tensile system is shown in Figure 5. The simulated longitudinal mode frequency of the whole resonant system was similar to the design frequency, the vibration node position distribution was reasonable, and the stress in the middle position of the tensile specimen was large, which basically met the design requirements.

**Figure 6:**
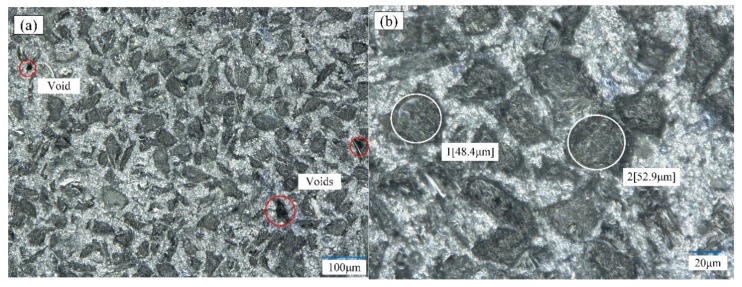
The observation of the surface morphology of the SiCp/Al composite; (**a**) Scanning electron microscopy overall surface topography; (**b**) Sic particle size.

**Figure 7:**
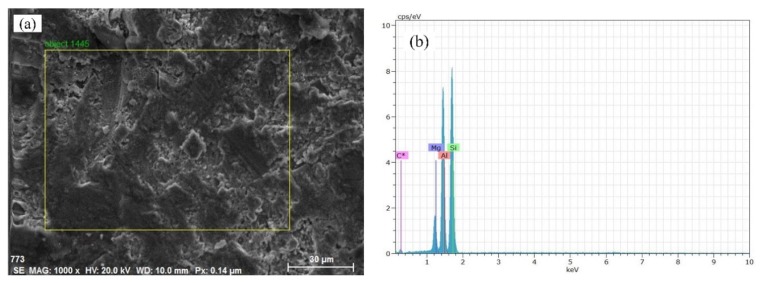
The energy spectrum analysis of the SiCp/Al composites; (**a**) Energy spectrum analysis block diagram; (**b**) SiCp/Al chemical compositions.

In this article, 70% SiCp/Al composites were prepared by powder metallurgy from Beijing Tianma Metal Processing Co., Ltd., and the porosity content was 6.2%. The Al matrix material is 7075 aluminum, and the chemical composition was analyzed via an energy dispersive spectrometer (EDS, INLA-ENERAGY 250), as shown in Table 5. The particle size of SiCp in the material was 40−50 μm. This phenomenon was observed under the Keyence Super Depth of Field VHX-2000 after intercepting a piece of material for grinding and polishing. The surface morphology of the SiCp/Al composites is shown in Figure 6. In Figure 6a, the gray portion is SiCp, and the white color is the aluminum matrix, while a small amount of black in the figure is the void formed by the falling off of the SiC particles. It can be seen from Figure 6b that the SiCp is similar in size and evenly distributed. The sample material is densely structured, while the interface between the SiC-reinforcing particles and the matrix is well bonded. The material was subjected to an energy spectrum analysis using a scanning electron microscope. The results are shown in Figure 7b, and the different chemical compositions were accounted for, as shown in Table 6.

## 3. Simulation, Experimental Results and Discussion

### 3.1. Ultrasonic Vibration Assisted Tensile ABAQUS Finite Element Simulation

**Figure 8:**
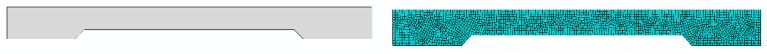
The two-dimensional axisymmetric model and meshing.

Since the sample is a rotational axisymmetric body, the tensile process is simplified to an axisymmetric two-dimensional analysis to simplify the solution process and shorten the calculation time, the model is shown in Figure 8. The material-related parameters are shown in Table 1.

Because of the high frequency of the ultrasonic vibration when the whole process of ultrasonic vibration is simulated, the stress and strain data points are too dense, and the calculation amount is too large to be realized. Therefore, the main simulation of this paper is the conventional tensile strength and intermittent vibration tensile strength of the high-part SiCp/Al composites’ tensile process. First, a conventional tensile simulation is performed, and ultrasonic vibration is applied after a certain period of time. The analysis time for the conventional tensile process was selected, according to the experiment, to be 45 s, and it was followed by the application of ultrasonic vibration for a duration of 20 cycles. In the conventional tensile stage, the application speed was 0.0167 mm/s. The intermittent vibration tensile stage involves superimposing ultrasonic vibration on the basis of conventional tensile strength; consequently, the total displacement during the ultrasonic vibration tensile strength is:

(4) S=νt+Asin(2πft) .

Derivatives from the above formulas are available:

(5) ν=ν+2πfAcos(2πft),

where ν is the initial velocity (average tensile rate), and A is the ultrasonic amplitude.

The ultrasonic vibration is applied via the velocity of the boundary condition while applying the amplitude by the ’Periodic’ method in the amplitude curve, where the angular frequency is 2πf, the initial time is 0, and the initial amplitude corresponds to the tensile rate: A=0,B=2πfA. The mesh uses an axisymmetric four-node unit with a meshing precision set to 1.

In order to explore the influence of each vibration parameter on the material deformation during the tensile process, the three-factor four-level tensile simulation scheme designed is shown in Table 7.

**Figure 9:**
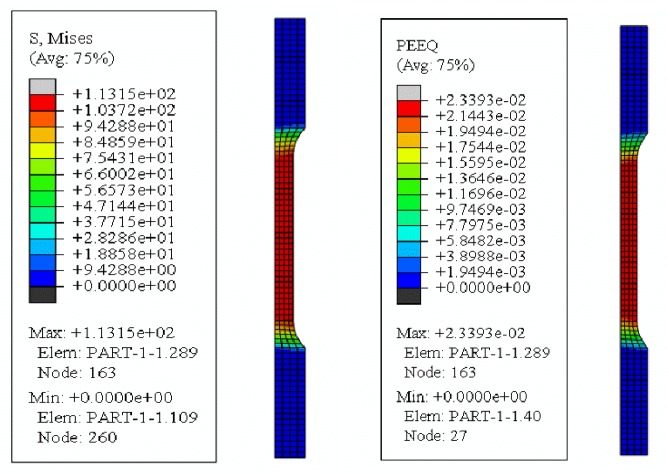
The conventional tensile stage simulation cloud.

**Figure 10:**
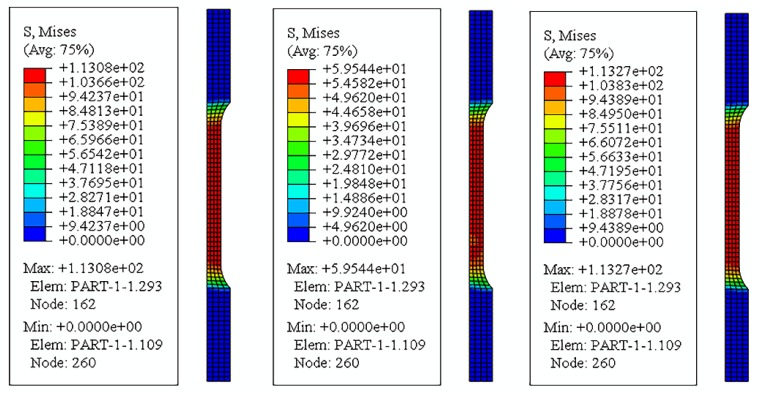
The ultrasonic vibration stage stress cloud.

Figure 9 is a graph of the stress and strain corresponding to the end of the conventional tensile phase. As shown in Figure 9, the maximum values of stress and strain are concentrated in the gauge length section of the sample, and the maximum stress at this time is 113 MPa, while the maximum strain is 0.0233. The intermittent vibration tensile strength is the application of ultrasonic vibration on the basis of the conventional tensile strength, taking an ultrasonic frequency of 20 kHz and an amplitude of 4 μm tensile process as an example. As shown in Figure 10, in an ultrasonic vibration period, the stress of the ultrasonic vibration gradually decreases from the zero-point time 113.08 MPa to the lowest point 59.5 MPa. When the direction of the velocity is upward, the material is stretched again, and the stress rises to a maximum point of 113.27 MPa. Thereafter, the stress state of the material circulates along the cosine period. Since the time for applying the ultrasonic vibration is very short, the strain of the material remains constant during the vibration phase.

**Figure 11:**
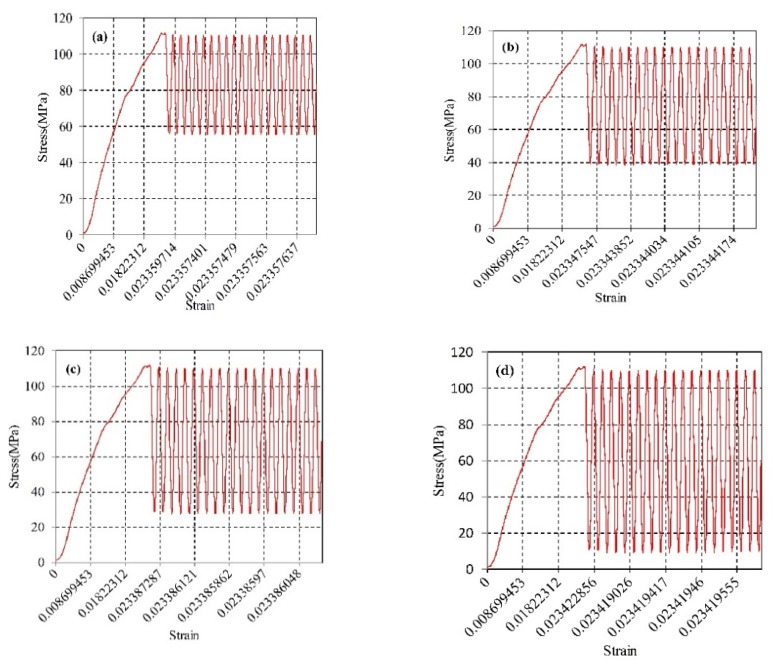
The simulation stress-strain curves under different amplitudes of the ultrasonic frequency 20 kHz: (**a**) Amplitude 4 μm; (**b**) Amplitude 5.2 μm; (**c**) Amplitude 6 μm; and (**d**) Amplitude 7.3 μm.

**Figure 12:**
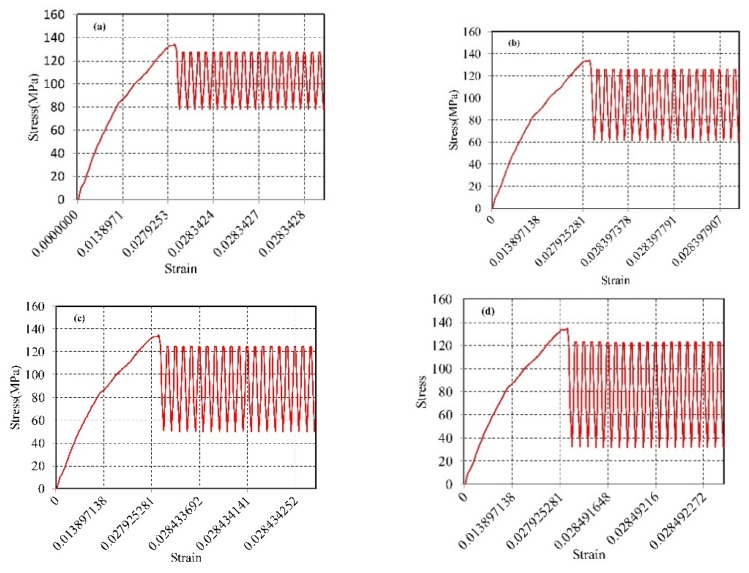
The simulation stress-strain curves under different amplitudes of the ultrasonic frequency 28 kHz, (**a**) Amplitude 3 μm; (**b**) Amplitude 4.8 μm; (**c**) Amplitude 6.3 μm; and (**d**) Amplitude 7.8 μm.

Figure 11 and Figure 12 are the simulated stress-strain curves for ultrasonic amplitudes of 20 kHz and 28 kHz, respectively. As shown in Figure 11 and Figure 12, the stress and strain increase linearly during the conventional tensile phase; when the ultrasonic vibration is applied, the stress drops to the lowest value, after which the curve tends to oscillate, and the peak value of the oscillation curve is lower than the initial value of the applied vibration. This indicates that the reduction in the material stress after the vibration application is the result of a combination of the softening effect and stress superposition effect. Furthermore, at a frequency of 28 kHz, the peak value of the oscillation curve decreases relative to the initial value by a higher amount than for the frequency at 20 kHz, which indicates that, as the frequency increases the magnitude of the stress reduction increases accordingly. At the same time, comparing the stress values of different amplitudes at the same frequency, the magnitude of the stress reduction increases with the increase of the amplitude.

### 3.2. Ultrasonic Vibration Tensile Experimental Verification

**Figure 13:**
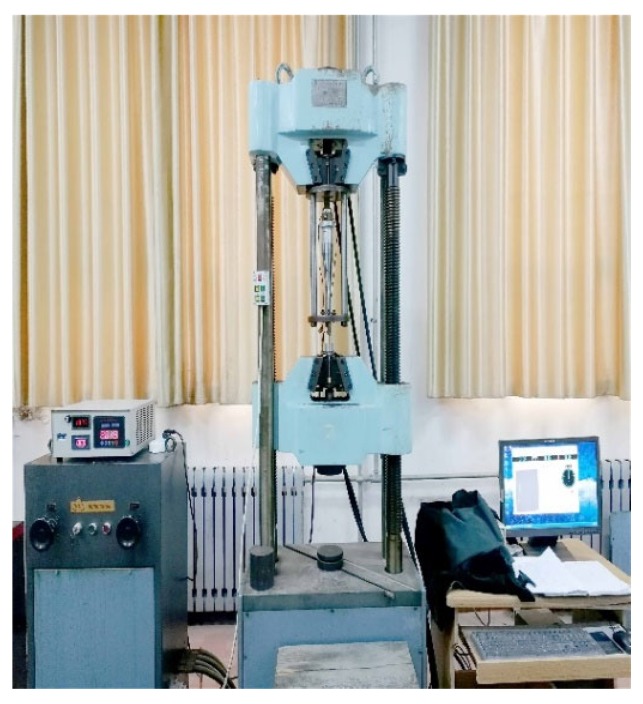
Schematic of the ultrasonic vibration tensile device.

**Figure 14:**
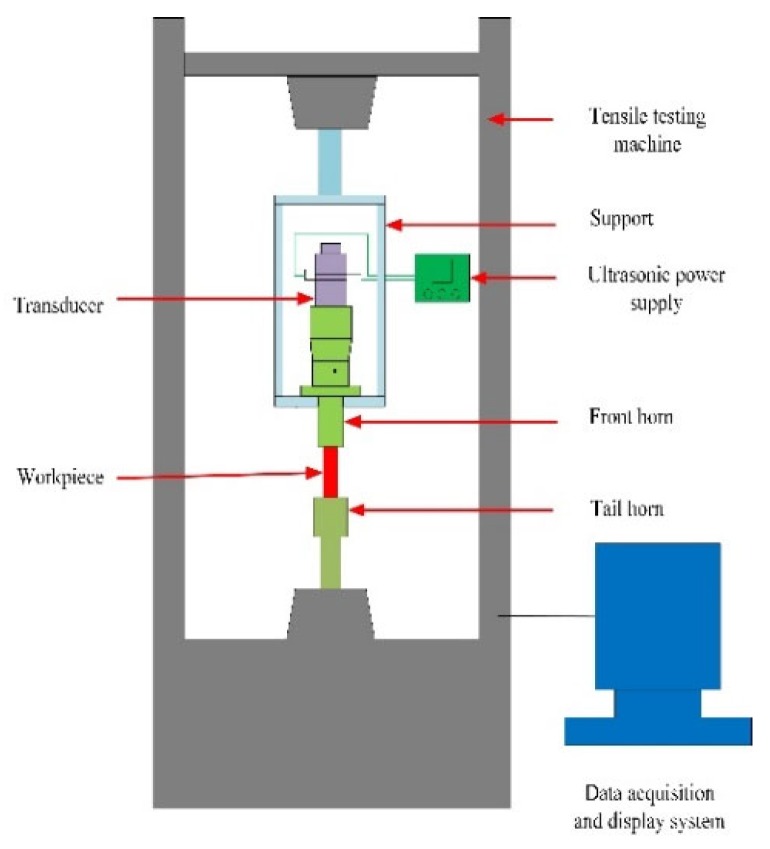
The ultrasonic tensile resonance system.

In order to apply ultrasonic vibration during the tensile process, an ultrasonic tensile device was designed based on the WAW-300kN electro-hydraulic servo universal testing machine manufactured by Tianshui Hongshan Testing Machine Company, as shown in Figure 13 and Figure 14. The SZ12 intelligent ultrasonic power supply produced by Hangzhou Success Ultrasonic Vibration Equipment Company is shown in Figure 13. The main parameters were a voltage of 220 V, a frequency of 50 Hz, an output power of 500 W, and an adjustable frequency range from 18 to 23 kHz and from 26 to 32 kHz.

The tensile experiment was carried out at a rate of 1 mm/min at room temperature. The samples were subjected to a conventional tensile strength: an ultrasonic vibration tensile strength at different frequencies, different amplitudes, and different durations. The tensile deformation measured by the software included not only the elongation of the sample but also the elastic deformation of the machine and the sliding of the clamping portion. Therefore, the application of ultrasound by load size can accurately determine the length of time of the ultrasound application. The common tensile material was shown to have a breaking load of 7 kN and a breaking strain of 0.05. It was determined that the ultrasonic wave was applied at a load of 3 kN and a strain of 0.025 until the load reached 5 kN, which was an intermittent vibration. The specific scheme is shown in Table 8.

**Figure 15:**
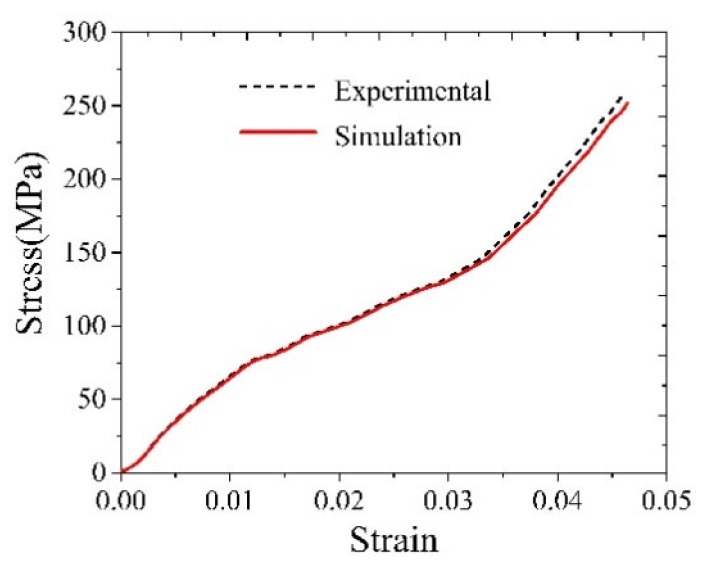
The experimental and finite element simulation stress-strain curve during conventional tensile strength.

Figure 15 shows the experimental and finite element simulation stress-strain curves for the conventional tensile strength. It can be seen from the figure that the error between the experimental and simulated stress-strain curves is small.

**Figure 16:**
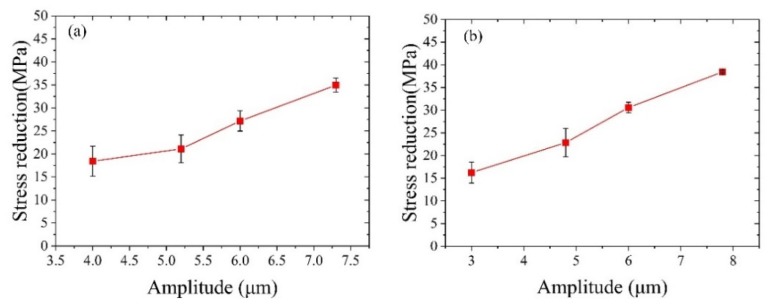
The magnitude of the stress reduction under different amplitudes of ultrasonic vibration: (**a**) 20 kHz, and (**b**) 28 KHz.

As shown in Figure 16, as the amplitude of the ultrasonic vibration increases, the magnitude of the stress gradually decreases. The analysis is as follows: As the ultrasonic amplitude increases, the Hall-Petch strengthening effect causes the material to enter the yielding stage in advance to promote the grain refinement stress. However, the larger the amplitude, the greater the energy, and the more obvious the softening effect. Thus, the softening effect of the major factor leads to an increase in the stress reduction. The magnitude of the stress reduction increases as the amplitude of the ultrasound increases, which is consistent with the previous simulation results.

**Figure 17:**
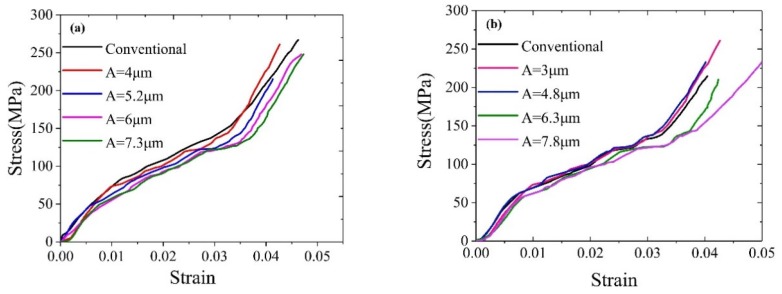
The experimental stress-strain curve under different amplitude conditions: (**a**) 20 kHz, and (**b**) 28 kHz.

Figure 17a shows the stress-strain curve of the sample at different amplitudes under intermittent vibration at a frequency of 20 kHz. The curves of different amplitudes were basically the same under a conventional tensile strength. The stress-strain curve of the material deviated significantly from the original trend following the application of ultrasound: at the same strain, the larger the amplitude is, the smaller is the stress and the greater is the stress reduction, which is the same as for the simulation results. Figure 17b, which is similar to the 20 KHz frequency trend, shows the experimental stress-strain curve of samples of different amplitudes under intermittent vibration at a frequency of 28 kHz.

**Figure 18:**
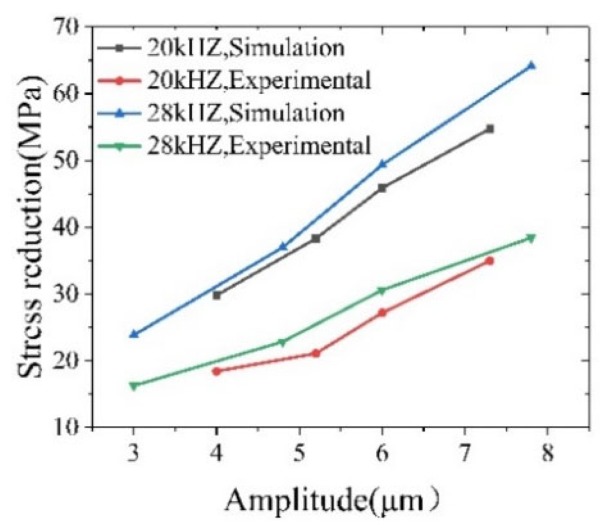
A comparison of the experimental and simulated values of the stress reduction.

Figure 18 shows the experimental and simulated values of the stress reduction amplitude at different frequencies and different amplitudes. The study shows that the experimental and simulation values are basically consistent with the changes in the ultrasonic amplitude and ultrasonic frequency. Under the same ultrasonic frequency, the simulation value of the stress reduction amplitude increases with the increase of the amplitude; with the increase of the ultrasonic frequency, the stress reduction amplitude increases slightly, which is consistent with the previous simulation results. The difference between the simulated and the experimental values in the figure is as follows: (1) The data sampling frequency of the testing machine is lower than the ultrasonic vibration frequency, and there is an acquisition error; (2) There is a loss in the ultrasonic energy transfer process, and the ultrasonic vibration acting on the sample is weakened; (3) The limitations of the simulation model cannot simulate the strengthening effect of the ultrasonic vibration on the material. The above reasons cause a certain degree of distortion in the simulation process, which results in some differences between the simulation results and the experimental results.

### 3.3. Simulation to Explore the Effect of Ultrasonic Parameters on the Magnitude of Stress Reduction

**Figure 19:**
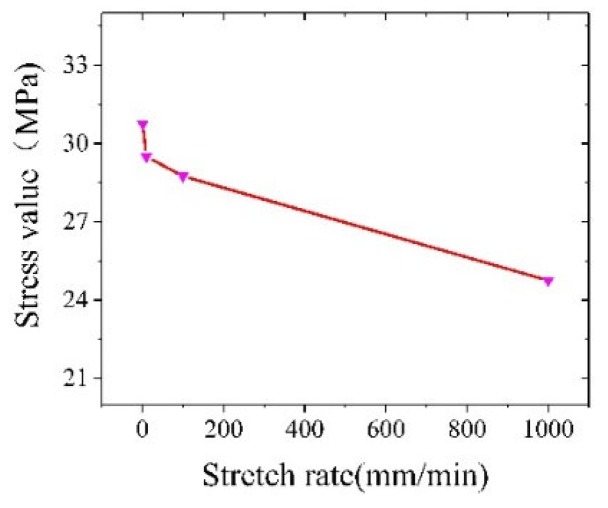
The effect of the tensile rate on the magnitude of the stress reduction.

As shown in Figure 19, the variation trend of the stress reduction amplitude under the conditions of an ultrasonic frequency of 20 kHz, amplitude of 4 μm and different tensile speeds were simulated. The magnitude of the stress reduction decreases as the tensile rate increases. Since the frequency and amplitude are constant values, the increase in the tensile rate causes the average flow stress to rise, so that the stress reduction amplitude decreases.

In order to explore the influence of the ultrasonic frequency, ultrasonic amplitude and tensile rate on the magnitude of the stress reduction, a three-factor and four-level orthogonal simulation experiment [24] was designed according to Table 6, and a simulation was carried out. The orthogonal simulation results are shown in Table 9:

For the orthogonal simulation results of Table 8, the intuitive analysis method was used to calculate the extreme difference of each factor, in order to determine the influence of the frequency, amplitude and tensile rate on the stress reduction amplitude. The intuitive analysis table of the stress reduction range is shown in Table 10.

It can be seen from Table 10 that the extremes of each factor are different, which indicates that the influence of the level change of each factor on the experimental results is different; additionally, the greater the difference, the greater the influence of the factor [24]. The analysis shows that the magnitude of the extreme difference of each factor is: $R_{A}>R_{\nu }>R_{f}$, indicating that the amplitude is the primary consideration in the experiment, followed by the tensile rate, while the frequency variation has the least influence on the magnitude of the stress reduction.

## 4. Conclusions

The finite element simulation of the ultrasonic vibration tensile strength was carried out for high-volume SiCp/Al composites. An ultrasonic vibration tensile composite dumbbell-shaped device was designed. The conventional tensile strength and intermittent vibration tensile strength were simulated by ABAQUS. The effects of the amplitude, frequency and tensile rate on the mechanical properties of the materials were discussed. According to the size of the load, the experiment on the conventional tensile strength, intermittent vibration tensile strength, and full vibration tensile strength is verified by simulation. The comparison shows that the experimental results are consistent with the simulation results. We have the following conclusions:

(1) In the conventional tensile stage, the simulation agrees with the experimental results. After the intermittent vibration simulation based on the conventional tensile strength, the peak value of the oscillation curve is lower than the initial value of the applied vibration. It is shown that after applying ultrasonic vibration, the softening effect and the stress superposition effect work together to cause a decrease in the material stress. At the same time, comparing the peak drop of the oscillation curves of different frequencies, we know that the magnitude of the stress reduction increases correspondingly with the increase of the frequency.
(2) The experimental value of the stress reduction range is consistent with the simulation value trend. However, due to the small sampling frequency, the ultrasonic transmission loss and the limitations of the simulation, we were unable to load the ultrasound enhancement result in numerical differences. Comparing the simulated value with the experimental value, it is found that the magnitude of the stress reduction is proportional to the amplitude under the same ultrasonic frequency.
(3) The tensile simulation of different tensile rates shows that the tensile rate is inversely proportional to the magnitude of the stress reduction. The orthogonal simulation and range analysis of the ultrasonic parameters show that the amplitude has the greatest influence on the stress reduction amplitude, that the tensile rate is second, and that the frequency has the least influence on the stress reduction amplitude.

## Figures and Tables

**Table 1:** Material-related parameters.

Material	Density $\rho /\left(kg\cdot m^{3}\right)$	Elastic Modulus $E/\left(kg\cdot mm^{-2}\right)$	Sound Speed c/(m/s)	Poisson’s Ratio v
45	7850	20,920	5170	0.3
SiCp/Al	3040	258,000	9212	0.3

**Table 2:** 1 stage horn parameters.

Sample Frequency	$D_{1}\left(\mathrm{mm}\right)$	$D_{2}\left(\mathrm{mm}\right)$	$L_{1}\left(\mathrm{mm}\right)$	$L_{2}\left(\mathrm{mm}\right)$	$M_{p}$
*f* = 20 kHZ	52	24	41	98	3.225
*f* = 28 kHZ	38	24	31	65	2.03

**Table 3:** 2 stage horn parameters.

Sample Frequency	$D_{1}\left(\mathrm{mm}\right)$	$D_{2}\left(\mathrm{mm}\right)$	$L_{1}\left(\mathrm{mm}\right)$	$L_{2}\left(\mathrm{mm}\right)$	$M_{p}$
*f* = 20 kHZ	24	16	64.5	64.5	2.25
*f* = 28 kHZ	24	16	46	46	2.25

**Table 4:** Sample size.

Sample Frequency	$r_{1}\left(\mathrm{mm}\right)$	$r_{2}\left(\mathrm{mm}\right)$	$x_{1}\left(\mathrm{mm}\right)$	$x_{2}\left(\mathrm{mm}\right)$	Total length(mm)
*f* = 20 kHZ	3	8	21.6	15	73.2
*f* = 28 kHZ	3	8	21.6	8	59.2

**Table 5:** The chemical composition of the 7075Al.

Element	Si	Mn	Ti	Cr	Cu	Mg	Zn	Fe	Al
Quality score %	0.40	0.30	0.20	0.21	1.5	2.3	5.6	0.50	margin

**Table 6:** The chemical composition of the SiCp/Al composite.

Element	Mg	O	Si	Al	Other
Quality score %	6.18	6.72	46.47	31.17	margin

**Table 7:** The ultrasonic vibration tensile simulation experimental scheme.

Factor	Level			
	1	2	3	4
*f* (kHz)	20	24	28	32
*A* (μm)	4	5	6	7
*v* (mm/min)	1	10	100	1000

**Table 8:** The tensile experimental design.

Numbering	*f* (kHz)	A (μm)	*v* (mm/min)	Vibration Application Method
P1	0	0	1	No vibration
U1	20	4	1	3.3–5 kN
U2	20	5.2	1	3.3–5 kN
U3	20	6	1	3.3–5 kN
U4	20	7.3	1	3.3–5 kN
U5	20	6	1	Full vibration
U6	28	3	1	3.3–5 kN
U7	28	4.8	1	3.3–5 kN
U8	28	6.3	1	3.3–5 kN
U9	28	7.8	1	3.3–5 kN
U10	28	6.3	1	Full vibration

**Table 9:** The orthogonal simulation results of the stress reduction amplitude.

Numbering	Frequency *f* (kHz)	Amplitude A (μm)	Tensile Rate *v* (mm/min)	Stress Reduction Δ*σ* (MPa)
1	20	4	1	30.75
2	20	5	10	36.51
3	20	6	100	42.33
4	20	7	1000	44.24
5	24	4	10	32.08
6	24	5	1	40.73
7	24	6	1000	39.27
8	24	7	100	53.19
9	28	4	100	31.63
10	28	5	1000	35.51
11	28	6	1	50.9
12	28	7	10	59.14
13	32	4	1000	25.53
14	32	5	100	38.37
15	32	6	10	52.17
16	32	7	1	61.13

**Table 10:** The intuitive analysis table of the stress reduction.

Numbering	Frequency *f* (kHz)	Amplitude A (μm)	tensile Rate *v* (mm/min)
k1	38.46	30	45.88
k2	41.32	37.78	44.98
k3	44.3	46.17	41.38
k4	44.3	54.25	36.14
Range	5.84	24.25	9.74
